# Characterization of the complete mitochondrial genome of *Saron marmoratus* (Hippolytidae, Decapoda) and its phylogenetic analysis

**DOI:** 10.1080/23802359.2020.1848474

**Published:** 2021-01-12

**Authors:** Yan Wang, Ling Zeng, Jing Wen, Xuyan Li, Yafen Huang, Yulin Sun, Juan Zhao

**Affiliations:** a Department of Scientific Research, Lingnan Normal University, Zhanjiang, PR China; b Department of Chemistry, Lingnan Normal University, Zhanjiang, PR China; c Department of Biology, Lingnan Normal University, Zhanjiang, PR China

**Keywords:** *Saron marmoratus*, mitochondrial genome, phylogenetic analysis, Hippolytidae

## Abstract

The complete mitochondrial genome of shrimp *Saron marmoratus* was obtained and characterized in this study. This complete mitochondrial genome is 16,330 bp in size and comprises 13 protein-coding genes (PCGs), two ribosomal RNA genes, and 22 transfer RNA genes. Fourteen genes were encoded by light strand and another 23 genes were encoded by heavy strand. The A + T content of the heavy-strand was 67.89%. Most PCGs had ATN as the start codon except ND4 initiated with GAT. Eleven PCGs terminated with a complete stop codon TAN, but two PCGs (*ND5* and *Cytb*) had an incomplete stop codon. The phylogenetic analysis suggested that Hippolytidae shrimp may be considered as the polyphyletic taxon. These results are useful for understanding the phylogenetic relationships and evolution of Hippolytidae shrimp.

Family Hippolytidae contains species of considerable economic interest in the Western Indian Ocean (Josileen 2013). *Saron marmoratus* (Olivier 1811), commonly known as the marbled shrimp, is a species of shrimp in the family Hippolytidae. Marbled shrimps are in high demand and good priced in the marine aquarium trade. It has a typical greenish to light brown in body color with whitish and yellowish speckled spots, and banded red and white legs. *S. marmoratus* spreads over the whole Indo-West-Pacific region and lives in caves, between rocks, and on coral reefs, near low depth coastal areas and in coastal ponds, between green algae (Sheibani-Tezerjiand and Sari 2007). This species has five zoeal stages and one megaropal stage before metamorphosing into the first juvenile stage under laboratory conditions (Yasuhiko and Naoki 2002). The mitochondrial genome is an effective tool for species identification, molecular taxonomy, and phylogenetic analyses (Galtier et al. 2009). Nowadays, only a few *S. marmoratus* mitochondrial DNA (mDNA) sequences such as 16 s and cox1 are found in GenBank. In this study, the complete mitochondrial genome of *S. marmoratus* was reported, which provides useful information for the phylogenetic analyses of *S. marmoratus*.

The specimen was collected from Shenzhen, Guangdong province, China (N22°35′, E114°31′) and deposited in the Zoological Herbarium, Lingnan Normal University (Acc. Number SC20200605-11). The muscle of *S. marmoratus* was fixed in 100% ethanol and stored at −20 °C. Approximately 30 mg of muscle tissue was used for mtDNA extraction with TIANamp Marine Animals DNA Kit (Tiangen, Beijing, China) according to the manufacturer’s specification. Quantity and integrity of the DNA samples were confirmed by 1% agarose gel and a 2100-Bioanalyzer (Agilent Technologies, Santa Clara, CA). Genomic DNA (500 ng) was sheared through sonication to an average insert size of 500 bp. Library preparation was performed with a NEB Next Ultra DNA Library Prep kit for Illumina (NEB, Ipswich, MA) according to the manufacture’s instruction. MtDNA was sequenced using the Illumina Hiseq Sequencing System (Illumina Inc., San Diego, CA). The clean data were acquired and assembled by the SPAdes and PRICE (Bankevich et al. 2012). BLAST (http://www.ncbi.nlm.nih.gov/BLAST/), ORFs finder (https://www.ncbi.nlm.nih.gov/orffinder/), and MITO (Bernt et al. 2013) were used to identify and annotated protein-coding genes (PCGs). MITO (Bernt et al. 2013) was used to identify tRNA genes. The complete 13 concatenated PCGs from 32 shrimps were downloaded from GenBank database. All of them were used to construct a phylogenetic tree which was performed using the maximum-likelihood (ML) method and the Kimura 2-parameter model implemented in the MEGA version 6.0 program with 1000 bootstrap replicates (Tamura et al. 2013).

The mitochondrial genome of *S. marmoratus* is 16,330 bp in length (GenBank accession number: MT795210) and containing the typical set of 13 PCGs, 22 tRNA, and 2 rRNA genes. The overall base composition of the heavy-strand was 37.68% A, 10.42% G, 21.70% C, and 30.21% T, with a high A + T content of 67.89%. Of the 37 genes, 23 genes were encoded by the heavy strand and 14 genes including 4 PCGs (*ND1*, *ND4*, *ND4L*, and *ND5*), 8 tRNA, and 2 rRNA were encoded by the light strand.

Most PCGs had ATN as the start codon except ND4 initiated with GAT. Eleven PCGs terminated with a complete stop codon TAN, but two PCGs (*ND5* and *Cytb*) had an incomplete stop codon (T–). The 16S and 12S rRNAs were 1332 bp (29.58% GC content) and 824 bp (28.88% GC content) in length, respectively. All tRNA genes were ranged from 64 to 70 bp in size and had the typical cloverleaf secondary structure. A non-coding region located between the 12S rRNA and tRNA-Ile genes was 985 bp in length.

*S. marmoratus*, that belongs to the Hippolytidae family is clustering with *Rhynchocinetes durbanensis* from the Rhynchocinetidae family. Remaining Hippolytidae species (*Lysmata amboinensis*, *Thor amboinensis*, and *Lebbus groenlandicus*) do not form a monophyletic group (Figure 1). This result suggests that Hippolytidae can be considered as polyphyletic taxon, which is same with the results of previous research (Terossi et al. 2017). This newly reported genome of *S. marmoratus* will be useful for the phylogenetic studies of *S. marmoratus.*

**Figure 1.**
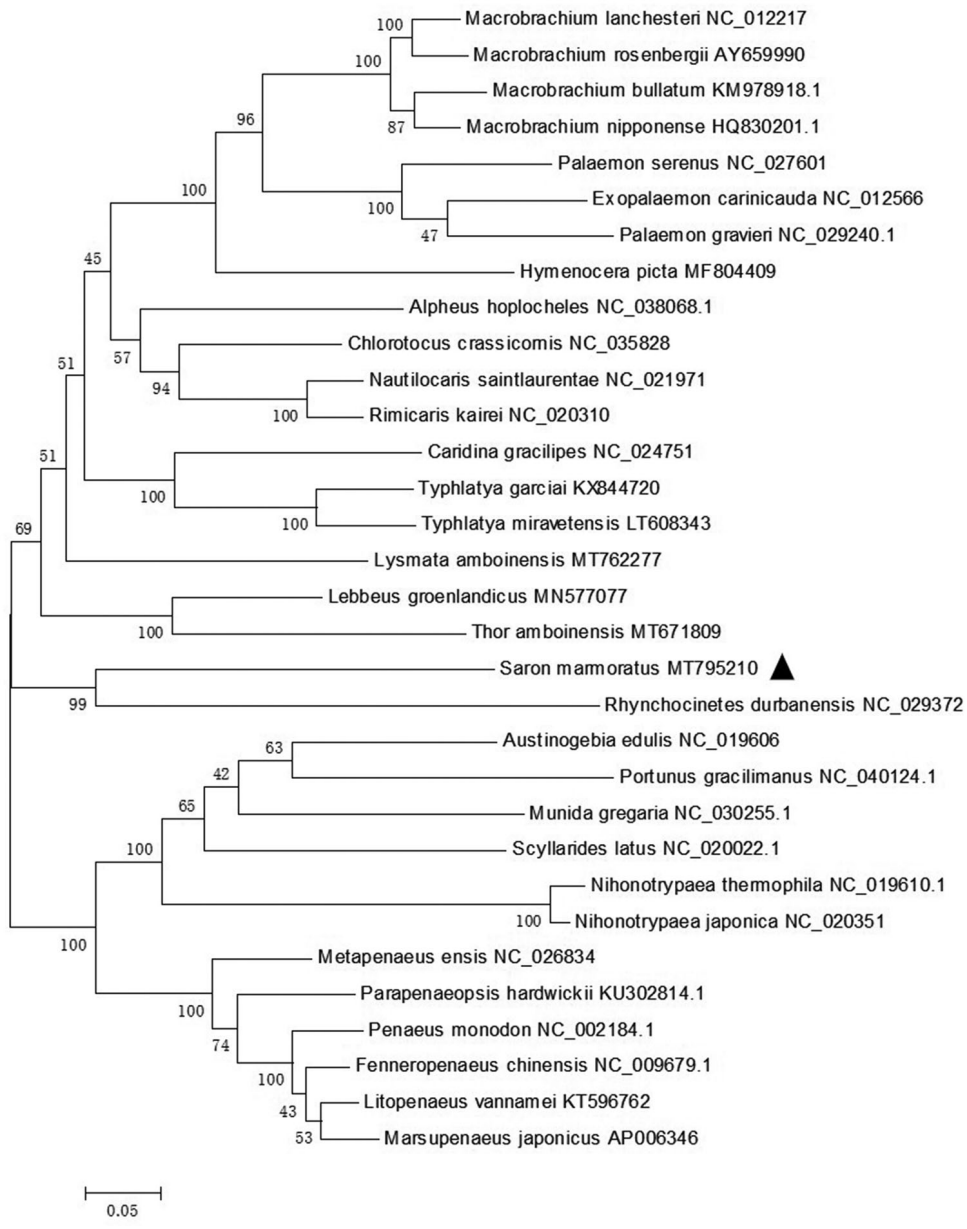
Phylogenetic tree of *S. marmoratus* and related species based on maximum-likelihood (ML) method.

## Data Availability

Mitogenome data supporting this study are openly available in GenBank at: https://www.ncbi.nlm.nih.gov/nuccore/MT795210. Associated BioProject, SRA, and BioSample accession numbers are http://www.ncbi.nlm.nih.gov/bioproject/671511, https://www.ncbi.nlm.nih.gov/sra/PRJNA671511, and https://www.ncbi.nlm.nih.gov/biosample/SAMN16533718, respectively.
